# Modulation of the PI3K/AKT/mTOR and AMPK/TSC2/mTOR Pathways by N-Acetyl-L-Cysteine as a Protector of Embryonic Bodies from the Toxic Effect of Methylmercury

**DOI:** 10.3390/brainsci16050542

**Published:** 2026-05-21

**Authors:** Júlia Leão Batista Simões, Geórgia de Carvalho Braga, Charles Elias Assmann, Margarete Dulce Bagatini

**Affiliations:** 1 Graduate Program in Biochemistry, Federal University of Santa Catarina, Florianópolis 88040-900, SC, Brazil; 2 Medical School, Federal University of Fronteira Sul, Chapecó 89815-899, SC, Brazil; 3 Graduate Program in Biological Sciences: Toxicological Biochemistry, Federal University of Santa Maria, Santa Maria 97105-900, RS, Brazil; 4 Graduate Program in Medical Sciences, Federal University of Fronteira Sul, Chapecó 89815-899, SC, Brazil

**Keywords:** methylmercury, developmental impairment, N-acetyl-L-cysteine, mTOR, oxidative stress, autophagy

## Abstract

**Highlights:**

**What are the main findings?**

• Harmful modulation of the mTOR pathway precipitates exacerbated autophagy and subsequent neurotoxicity.
• The oxidative stress inherent in MeHg neurotoxicity regulates neuronal autophagy via an mTOR-dependent mechanism.

**What are the implications of the main findings?**

• NAC provides neuroprotection by restoring mTOR signaling homeostasis and facilitating the systemic excretion of MeHg–thiol complexes.
• Therapeutic intervention with NAC mitigates the impact of MeHg-induced neuronal toxicity.

**Abstract:**

Methylmercury (MeHg) is a potent environmental contaminant primarily ingested through seafood consumption. Gestational exposure induces profound neurological and developmental deficits in the fetus that often persist throughout childhood. This developmental vulnerability arises from the immature state of the blood–brain barrier and a limited endogenous antioxidant capacity in the developing CNS. Postnatal exposure via breastfeeding further compromises neurodevelopment, specifically impairing visuospatial processing and memory. While fetal and placental mercury accumulation correlates with gestational age, the specific mechanisms of transplacental transport remain poorly defined. Mechanistically, MeHg predominantly accumulates in fetal renal tissue, followed by the brain and liver. This review aims to elucidate MeHg-induced oxidative stress and autophagic collapse mediated by the PI3K/AKT/mTOR and AMPK/TSC2/mTOR pathways. Furthermore, we evaluate neuroprotective candidates, specifically N-acetyl-L-cysteine (NAC) and CCL chemokine modulation, as strategies to mitigate fetal impairment and the associated cellular damage.

## 1. Introduction

As a potent organic contaminant, methylmercury (MeHg) bioaccumulates in aquatic food chains and reaches critical concentrations in humans primarily through seafood consumption [1]. Driven by biomagnification, MeHg levels can induce severe clinical symptoms in the central nervous system (CNS) [1,2]. Its pathological potential is largely attributed to its capacity to traverse the blood–brain barrier (BBB) and the placental barrier [2]. This poses a significant threat to the developing fetus, where MeHg exposure precipitates diverse complications, including impaired neurological development, congenital deformities, and preterm birth [3,4]. The severity of these impacts is dictated by exposure levels and the developmental stage of the brain, with the fetal, embryonic, and neonatal stages exhibiting extreme vulnerability [2].

While the mechanisms underlying MeHg-induced fetal impairment remain partially elucidated, current evidence highlights the role of reactive oxygen species (ROS) formation and the induction of autophagy [5,6]. Specifically, the inhibition of the mammalian target of rapamycin (mTOR)—a central regulator of cellular metabolism and the cell cycle—has been proposed as a primary pathological pathway [6,7]. Given that mTOR dysregulation is a hallmark of MeHg-induced developmental impairment, identifying potent pharmacological modulators is a priority for mitigating long-term fetal deficits.

In this context, N-acetyl-L-cysteine (NAC) has emerged as a multifaceted neuroprotective agent capable of modulating the PI3K/AKT/mTOR and AMPK/TSC2/mTOR axes [7]. Beyond its established antioxidant properties and ability to cross the BBB, NAC facilitates the replenishment of intracellular glutathione (GSH) pools and modulates glutamatergic signaling [8]. These mechanisms have demonstrated efficacy in mitigating neuronal damage induced by heavy metals [8]. The implementation of human cerebral organoids (hCOs) allows for more physiologically relevant modeling of neurodevelopment, demonstrating that AKT/mTOR dysregulation impairs neuronal differentiation and synaptic homeostasis [9].

Moving beyond the traditional focus on MeHg binding to sulfhydryl groups and subsequent cytoskeletal disruption [4,10], this review proposes a novel ‘signaling rescue’ model. We focus on the role of NAC as a molecular bridge to restore the equilibrium between mTORC1-mediated growth and AMPK-driven autophagic flux [7]. Thus, this work aims to elucidate the role of mTOR signaling in MeHg contamination and explore NAC as a potential pharmacological therapy to enhance the clinical management of MeHg-induced brain impairment.

## 2. Physiological Significance of mTOR Signaling in Neurodevelopment

The mammalian target of rapamycin (mTOR) constitutes a pivotal signaling hub that integrates extracellular stimuli—including nutrient availability, growth factors, and energy status—to regulate cellular metabolism and proteostasis [11,12]. During neurodevelopment, the canonical PI3K/AKT/mTORC1 axis serves as a critical driver of anabolic processes. Activation of phosphoinositide 3-kinase (PI3K) facilitates the recruitment and phosphorylation of Akt, which subsequently antagonizes the Tuberous Sclerosis Complex (TSC) to promote mTORC1-mediated protein synthesis, lipogenesis, and ribosomal biogenesis [7,13]. This axis is functionally essential for the proliferation of neural stem cell populations and the precise execution of synaptogenesis [9,11].

Complementing this growth-oriented signaling, the AMPK/TSC2/mTORC1 pathway acts as a homeostatic energy sensor [14,15]. Under conditions of metabolic stress or ATP depletion, the adenosine monophosphate-activated protein kinase (AMPK) directly phosphorylates TSC2, thereby repressing mTORC1 and initiating autophagic flux [7,14]. This mechanism is vital for maintaining cellular integrity through the clearance of damaged organelles and protein aggregates during neuronal maturation [15,16]. Concurrently, mTORC2 regulates the actin cytoskeleton and maintains apical–basal polarity, which is a prerequisite for organized neuronal migration within the developing cortical laminae [17,18].

The high metabolic demands and the reliance of these pathways on redox-sensitive thiol groups establish a state of inherent physiological vulnerability. In the context of environmental neurotoxicants, this signaling nexus becomes a primary target for biochemical disruption. Consequently, the hijacking of these specific regulatory nodes by methylmercury leads to a systematic failure of cellular homeostasis, the mechanisms of which are elucidated in the subsequent analysis of MeHg-induced molecular crosstalk.

## 3. Molecular Crosstalk: MeHg-Induced mTOR Inhibition and NAC-Mediated Rescue

The neurotoxic impact of MeHg is non-uniform across the CNS, as the hippocampus and cerebral cortex exhibit divergent pathological signatures. While prenatal exposure primarily disrupts neuronal migration and proliferation due to the high sensitivity of neural stem cells [2,19], postnatal exposure via breastfeeding tends to impact synaptogenesis and glial maturation [20]. These temporal differences likely stem from the maturation state of the BBB and the ontogeny of organic anion transporters, such as Oat1 [2,21].

In the developing cortex, MeHg disrupts neuronal migration and apical polarity, leading to disorganized laminar structures [18]. Conversely, the hippocampus—specifically the dentate gyrus—demonstrates higher rates of apoptosis and impaired neurogenesis [22]. This regional vulnerability is driven by differential Oat1 expression and baseline PI3K/Akt activity. Specifically, the hippocampus is susceptible to autophagic collapse due to limited antioxidant capacity and high metabolic demands during development [7,23].

Beyond oxidative stress, MeHg disrupts cytoskeletal dynamics by altering microtubule stability, subsequently impairing neuronal migration and laminar organization [18]. Additionally, its high affinity for sulfhydryl enzymes inactivates critical metabolic activities [4,24], while its ability to modify cellular DNA further exacerbates neurological impairment [24,25]. Recent epigenetic investigations reveal that MeHg exposure induces site-specific DNA hypermethylation of the mTOR promoter and alters histone acetylation patterns in fetal cortical neurons [7,23].

### 3.1. Convergence of Signaling Dysregulation

MeHg exposure triggers a sophisticated dual insult to the mTOR axis that effectively dismantles cellular homeostasis [7,17]. Primarily, MeHg inhibits the mTORC1 complex, an event that precipitates pathological autophagy through the dephosphorylation of ULK1 [7,15]. Simultaneously, the toxin disrupts the mTORC2 complex, characterized by impaired Akt phosphorylation at the Ser473 site, which ultimately catalyzes cytoskeletal collapse and the loss of neuronal polarity [7,17].

Beyond this canonical pathway, recent evidence demonstrates that MeHg also induces mTOR-independent autophagy through the activation of the JNK/Vps34 complex, promoting the pathological accumulation of autophagosomes and subsequent lysosomal degradation failure [26]. Concurrently, the accumulation of MeHg-induced ROS and the depletion of intracellular ATP pools activate the AMPK/TSC2 pathway [7,15]. This activation further represses mTORC1, inducing a state of “biochemical starvation” that shifts autophagic flux from a cytoprotective process toward a pathological, cytotoxic phenotype [7,15]. By attacking these regulatory nodes from both ends—suppressing survival signaling while mimicking energy depletion—MeHg creates a metabolic crossroads that favors neuronal death over adaptation.

### 3.2. The Hierarchical Cascade of Neurotoxicity

The interplay between ROS, epigenetics, and autophagy follows a defined temporal and causal sequence that dictates the progression of MeHg-induced damage [7,27]. This hierarchy begins with a primary biochemical trigger, where ROS generation acts as the immediate initiator [7,28]. MeHg’s high affinity for sulfhydryl groups leads to the rapid depletion of glutathione (GSH) and the inhibition of antioxidant enzymes, creating an oxidative environment that acts as a “molecular brake” on survival signaling [7,28]. This leads to the secondary regulatory layer, the “epigenetic lock,” where ROS and MeHg exposure upregulate DNMT3a, catalyzing the hypermethylation of the mTOR promoter region [27,29]. This hypermethylation establishes a sustained suppression of the pathway, effectively creating a “pro-apoptotic cellular memory” that persists even after the initial chemical exposure has subsided [27]. Finally, the tertiary effector is functional; the combined pressure of oxidative stress and epigenetic silencing forces autophagic flux from a basal, homeostatic state into a pathological, cytotoxic phenotype [7,15]. This autophagic collapse serves as the final executor of neuronal cell death, marking the end stage of the hierarchical collapse [26].

### 3.3. The Mechanistic Rescue by NAC

In this context, NAC therapy provides neuroprotection through a multifaceted restoration of these specific signaling nodes [7,8]. By neutralizing ROS, NAC effectively eliminates the “molecular brake” on the mTOR pathway, while simultaneously stabilizing PI3K/Akt phosphorylation to antagonize the overactivation of GSK-3β [13,28].

Beyond direct antioxidant action, NAC facilitates the sequestration of mercuric ions into nontoxic complexes, preventing further enzymatic inactivation and limiting the chemical’s ability to trigger the initial biochemical insult [22]. A critical dimension of this metabolic rescue involves the preservation of mitochondrial integrity; as MeHg exposure triggers excessive mitophagy mediated by the AMPK/mTOR axis due to a drop in membrane potential, NAC intervention acts to stabilize these organelles and prevent their premature degradation [30].

**Figure 1:**
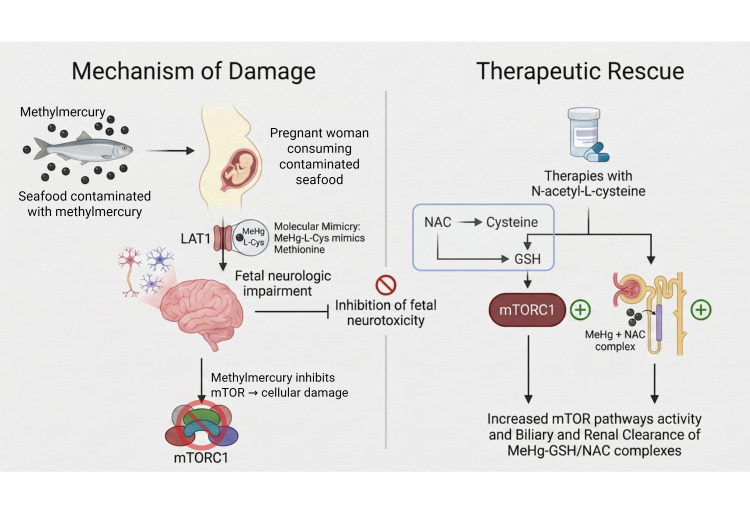
Mechanistic integration of methylmercury (MeHg)-induced neurotoxicity and therapeutic rescue mediated by N-acetyl-L-cysteine (NAC). Consumption of seafood contaminated with MeHg during pregnancy allows the formation of the MeHg-L-cysteine complex, which mimics methionine and crosses the placental barrier through the LAT1 transporter, promoting fetal neurological impairment. In the nervous system, MeHg induces oxidative stress and inhibits the mTORC1 pathway, resulting in cellular damage and neurotoxicity. As a therapeutic strategy, NAC acts as a precursor of cysteine and glutathione (GSH), restoring redox balance and favoring the reactivation of mTORC1 signaling. Furthermore, the formation of MeHg-GSH/NAC complexes facilitates the biliary and renal clearance of the metal, reducing its toxicity and contributing to fetal neuronal protection. Created with BioRender.com.

Furthermore, the convergence of metabolic stressors catalyzes a strategic metabolic shift toward GDH upregulation and enhanced fatty acid oxidation, transitions that are supported by NAC to restore mitochondrial bioenergetics and enhance neuronal resilience [30,31]. Ultimately, this signaling rescue provides a comprehensive intervention: it neutralizes the primary ROS trigger, potentially reverses the ‘epigenetic lock’ through promoter demethylation, and restores the homeostatic signaling balance necessary for neuronal survival, a mechanistic integration summarized in Figure 1 [7,29].

## 4. Therapeutic Potential of N-Acetyl-L-Cysteine in Fetal Methylmercury Exposure

The neuroprotective efficacy of NAC is primarily driven by its role as a thiol donor. By providing a source of sulfhydryl (-SH) groups, NAC facilitates the replenishment of intracellular GSH pools and enhances detoxification through increased glutathione-S-transferase activity [28,32].

Beyond classical ROS scavenging, NAC functions as a critical mediator of metabolic rescue by targeting mitochondrial enzymes. Electrophilic stressors induced by MeHg are known to inactivate glutamate dehydrogenase (GDH) by promoting intermolecular disulfide cross-linking at sensitive cysteine residues [31]. This enzymatic blockade stalls the Tricarboxylic Acid (TCA) cycle and depletes the precursor pools for GSH biosynthesis. NAC restores mitochondrial bioenergetics by reducing these disulfide cross-links, thereby reactivating key TCA cycle intermediates [31]. This transition from simple antioxidant defense to active metabolic restoration is fundamental for neuronal resilience during critical developmental windows.

**Figure 2:**
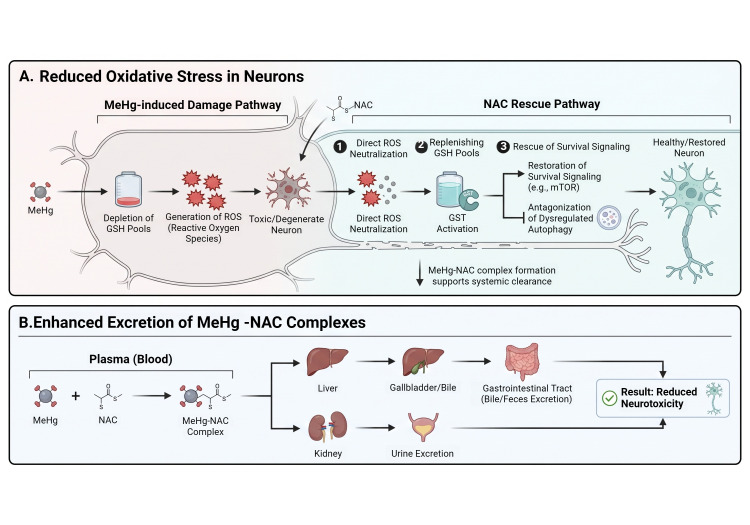
N-acetyl-L-cysteine (NAC) effects in MeHg neurotoxicity. Therapy with NAC acts through multiple protective pathways beyond direct ROS scavenging. In the intracellular compartment, NAC restores redox homeostasis and provides neuroprotection by preventing the hypermethylation of defensive gene promoters, such as Nrf2, thereby facilitating their epigenetic reactivation. Crucially, NAC acts as a biochemical switch to restore metabolic flux by reversing the electrophilic inactivation of glutamate dehydrogenase (GDH), desulfurizing inactive cross-linked protein complexes. These actions diminish neurologic damage by reinstating mitochondrial bioenergetics and survival signaling [29,31]. Created with BioRender.com.

This intervention is critical because MeHg actively undermines cellular defenses by impairing cystine uptake and depleting GSH content, a process that manifests as elevated malondialdehyde (MDA) levels and significantly reduced glutathione peroxidase (GSH-Px) activity [33,34,35,36]. Beyond its antioxidant capacity, NAC promotes MeHg detoxification by binding the organic–metal to facilitate its systemic expulsion [37]. As illustrated in Figure 2, this therapeutic impact is dual-layered: it provides a robust intracellular defense by restoring GDH activity and survival signaling, while simultaneously promoting the systemic expulsion of MeHg-NAC complexes through the biliary and renal pathways [31,37].

### 4.1. Clinical Transition and Dosage Optimization in Perinatal Models

Applying NAC as a neuroprotector requires a precise understanding of its therapeutic window in developing organisms. While high bolus doses are standard for adult acetaminophen poisoning, neonatal models suggest that excessive concentrations can be counterproductive. For instance, 200 mg/kg doses in P7 rats have been shown to reduce hippocampal DNA synthesis [22]. Conversely, repeated low-dose administration (10 mg/kg) maintains neuroprotective efficacy without metabolic interference. This suggests that a “low-dose, high-frequency” approach may be superior for perinatal exposure to avoid pathway saturation and the accumulation of prooxidant metabolites, such as S-nitroso-N-acetylcysteine (SNOAC) [38,39].

### 4.2. Modulation of Autophagy and Systemic Excretion

NAC effectively antagonizes MeHg-induced pathological autophagy by restoring signaling homeostasis within the developing CNS. Recent studies confirm that NAC alleviates MeHg-induced cytotoxicity in astrocytes and inhibits Purkinje cell loss in mice [40,41]. By normalizing the PI3K/Akt and AMPK/TSC2 axes, NAC pretreatment reduces the expression of pro-autophagy proteins that otherwise lead to irreversible neuronal damage [13,42,43,44,45,46]. In vivo evidence demonstrates that NAC prevents hippocampal cell loss and acute neurotoxicity even when brain MeHg levels decrease only marginally. This suggests that NAC-MeHg complexes may sequester mercuric ions in nontoxic forms or attenuate toxicity despite the limited excretion capacity of pre-weaned organisms [21,22,47,48,49,50].

As illustrated in Figure 2, the therapeutic impact of NAC is dual-layered: it provides a robust intracellular defense by restoring survival signaling, while simultaneously promoting the systemic expulsion of MeHg-NAC complexes. In contrast to the neonatal context, NAC in adults facilitates rapid urinary excretion of MeHg by forming stable complexes that are readily processed for elimination [28]. The strategic role of NAC as a molecular bridge for metabolic rescue is further supported by its efficacy in reversing the cytotoxic profiles of potent electrophiles in simpler eukaryotic systems. In *S. cerevisiae* models, the severe oxidative damage, mitochondrial membrane depolarization, and cell cycle arrest induced by GDH inhibition are fully restored upon NAC supplementation, which replenishes the cellular thiol pool and reactivates essential metabolic nodes [31].

### 4.3. Translational Gaps and Clinical Safety

Despite promising animal data, several critical translational gaps prevent the immediate clinical implementation of NAC for gestational MeHg exposure. A primary unknown is the exact kinetic behavior of NAC-MeHg complexes during maternal–fetal transfer. While NAC facilitates systemic expulsion in adult models, some evidence suggests it could theoretically act as a molecular transporter of mercury depending on the timing of administration and the maturational state of the placental barrier [51,52]. Furthermore, the lack of controlled clinical trials in pregnant populations creates a significant ethical and pharmacological hurdle for establishing standardized safety protocols.

Most current evidence relies on simpler eukaryotic systems or rodent models, which may not fully replicate the complex physiological responses of the human placenta or the extended gestational window in humans. Consequently, future research utilizing human organoid models is essential to bridge these gaps and functionally validate the “signaling rescue” observed in laboratory settings [22,51,52]. A comprehensive summary of the signaling alterations induced by MeHg across various experimental models, along with the restorative effects of NAC, is consolidated in Table 1.

## 5. Concluding Remarks and Future Perspectives

Current evidence confirms that therapy with NAC significantly improves clinical outcomes in cases of MeHg contamination by providing strategic neuroprotective benefits during the perinatal period. As MeHg profoundly dysregulates the PI3K/Akt/mTOR and AMPK/TSC2/mTOR signaling axes, targeted pharmacological interventions aimed at these specific nodes appear highly efficacious. However, further in vivo research remains essential to establish standardized protocols and optimal therapeutic concentrations for immature organisms, where the modulation of MeHg toxicity differs significantly from adult models. Future investigations should utilize advanced genetic tools, such as CRISPR-Cas9 or specific kinase inhibitors, to confirm the causal role of the PI3K/Akt/GSK-3β axis in MeHg-induced pathology. Beyond these genetic approaches, the investigation of selective mitophagy via the PINK1/Parkin pathway and the use of mitochondrial-targeted ROS scavengers, such as Mito-TEMPO, emerge as promising strategies to mitigate developmental defects.

Furthermore, emerging research suggests that the persistent suppression of defensive pathways may be governed by an “epigenetic lock” characterized by DNMT3a-mediated hypermethylation. Future therapeutic strategies should investigate the potential of antioxidants and specific bioactive compounds to reverse this epigenetic memory through promoter CpG demethylation of pathways like Nrf2. The integration of these treatments into human organoid models may bridge the gap between mechanistic research and definitive clinical application, functionally validating whether targeted genetic restoration of the GCL-mTOR node is sufficient to drive the adaptive rescue responses and metabolic resilience necessary to abrogate neurotoxicity. Additionally, the development of brain-targeted prodrugs with enhanced lipophilicity is required to overcome the current limitations of CNS penetration. Investigating synergies between NAC and epigenetic modulators may offer a multifaceted approach to reversing the long-term neurodevelopmental consequences of gestational exposure. Given that fish consumption remains a vital source of neuroprotective nutrients, the strategic use of NAC or related pharmacological compounds could significantly mitigate the public health risks associated with mercury-contaminated seafood ingestion.

## Figures and Tables

**Table 1:** Summary of MeHg-induced signaling alterations and NAC intervention.

Pathway/Target	Model	Effect of MeHg	Effect of NAC	Reference
PI3K/Akt/mTOR	Rat Cortex	↓ Phosphorylation	↑ Restoration	[7]
AMPK/TSC2	Human Stem Cells	↑ Activation	↓ Suppression	[46]
Autophagy (LC3-II)	Primary Astrocytes	↑ Flux	↓ Normalization	[40]
GSH Levels	Fetal Brain	↓ Depletion	↑ Repletion	[22]

## Data Availability

Not applicable.

## Abbreviations

The following abbreviations are used in this manuscript:

AKT	Protein kinase B
AMP	Adenosine monophosphate
AMPK	AMP-activated protein kinase
ATG1	Autophagy-related protein 1
ATG13	Autophagy-related protein 13
ATP	Adenosine triphosphate
BAX	Bcl-2-associated X protein
BBB	Blood–brain barrier
CAT	Catalase
DMPS	2,3-dimercapto-1-propanesulfonate
DMSA	Meso-2,3-dimercaptosuccinic acid
DNA	Deoxyribonucleic acid
GCL	Glutamate–cysteine ligase
GDH	Glutamate dehydrogenase
GSH	Glutathione
GSH-Px	Glutathione peroxidase
GSK-3β	Glycogen synthase kinase-3 beta
LC3	Microtubule-associated protein 1 light chain 3
MDA	Malondialdehyde
MeHg	Methylmercury
Mrp2/Abcc2	Multidrug resistance-associated protein 2
mTOR	Mammalian target of rapamycin
mTORC1	mTOR complex 1
mTORC2	mTOR complex 2
NAC	N-acetyl-L-cysteine
NRF2	Nuclear factor erythroid 2-related factor 2
Oat1	Organic anion transporter 1
PI3K	Phosphoinositide 3-kinase
Prx/Trx	Peroxiredoxin/thioredoxin system
ROS	Reactive oxygen species
SOD	Superoxide dismutase
SNOAC	S-nitroso-N-acetylcysteine
TSC2	Tuberous sclerosis complex 2
ULK1	Unc-51-like autophagy-activating kinase 1

## References

[1] Ni L., Wei Y., Pan J., Li X., Xu B., Deng Y., Yang T., Liu W. (2022). Shedding new light on methylmercury-induced neurotoxicity through the crosstalk between autophagy and apoptosis. Toxicol. Lett..

[2] Fujimura M., Usuki F. (2022). Cellular Conditions Responsible for Methylmercury-Mediated Neurotoxicity. Int. J. Mol. Sci..

[3] Hong Y.S., Kim Y.M., Lee K.E. (2012). Methylmercury exposure and health effects. J. Prev. Med. Public Health.

[4] Myers G.J., Davidson P.W. (1998). Prenatal methylmercury exposure and children: Neurologic, developmental, and behavioral research. Environ. Health Perspect..

[5] Novo J.P., Martins B., Raposo R.S., Pereira F.C., Oriá R.B., Malva J.O., Fontes-Ribeiro C. (2021). Cellular and Molecular Mechanisms Mediating Methylmercury Neurotoxicity and Neuroinflammation. Int. J. Mol. Sci..

[6] Wei Y., Ni L., Pan J., Li X., Xu B., Deng Y., Yang T., Liu W. (2021). The Roles of Oxidative Stress in Regulating Autophagy in Methylmercury-induced Neurotoxicity. Neuroscience.

[7] Wei Y., Ni L., Pan J., Li X., Deng Y., Xu B., Yang T., Sun J., Liu W. (2023). Methylmercury promotes oxidative stress and autophagy in rat cerebral cortex: Involvement of PI3K/AKT/mTOR or AMPK/TSC2/mTOR pathways and attenuation by N-acetyl-L-cysteine. Neurotoxicol. Teratol..

[8] Raghu G., Berk M., Campochiaro P.A., Jaeschke H., Marenzi G., Richeldi L., Wen F.-Q., Nicoletti F., Calverley P.M.A. (2021). The Multifaceted Therapeutic Role of N-Acetylcysteine (NAC) in Disorders Characterized by Oxidative Stress. Curr. Neuropharmacol..

[9] Chalkiadaki K., Statoulla E., Zafeiri M., Voudouri G., Amvrosiadis T., Typou A., Theodoridou N., Moschovas D., Avgeropoulos A., Samiotaki M. (2024). GABA/Glutamate Neuron Differentiation Imbalance and Increased AKT/mTOR Signaling in CNTNAP2-/- Cerebral Organoids. Biol. Psychiatry Glob. Open Sci..

[10] Choi B.H., Lapham L.W., Amin-Zaki L., Saleem T. (1978). Abnormal neuronal migration, deranged cerebral cortical organization, and diffuse white matter astrocytosis of human fetal brain: A major effect of methylmercury poisoning in utero. J. Neuropathol. Exp. Neurol..

[11] Paquette A.J., Gieske M.C., Kula-Eversole E., Williams R.L. (2018). mTOR signaling in neurodevelopment and its role in neurodevelopmental disorders. Dev. Biol..

[12] Kim J., Guan K.L. (2019). mTOR as a central hub of nutrient signalling and cell growth. Nat. Cell Biol..

[13] Fang S., Wan X., Zou X., Sun S., Hao X., Liang C., Zhang Z., Zhang F., Sun B., Li H. (2021). Arsenic trioxide induces macrophage autophagy and atheroprotection by regulating ROS-dependent TFEB nuclear translocation and AKT/mTOR pathway. Cell Death Dis..

[14] Gwinn D.M., Shackelford D.B., Egan D.F., Mihaylova M.M., Mery A., Vasquez D.S. (2008). AMPK phosphorylation of raptor mediates a metabolic checkpoint. Mol. Cell.

[15] Alers S., Löffler A.S., Wesselborg S., Stork B. (2012). Role of AMPK-mTOR-Ulk1/2 in the regulation of autophagy: Cross talk, shortcuts, and feedbacks. Mol. Cell. Biol..

[16] Al-Bari M.A.A., Xu P. (2020). Molecular regulation of autophagy and its role in neurodevelopmental and neurodegenerative diseases. Neural Regen. Res..

[17] Pan Z., Zhang H., Dokudovskaya S. (2023). The Role of mTORC1 Pathway and Autophagy in Resistance to Platinum-Based Chemotherapeutics. Int. J. Mol. Sci..

[18] Castoldi A.F., Coccini T., Manzo L. (2003). Neurotoxic and molecular effects of methylmercury in humans. Rev. Environ. Health.

[19] Buzanska L., Sypecka J., Nerini-Molteni S., Compagnoni A., Hogberg H.T., del Torchio R., Domanska-Janik K., Zimmer J., Coecke S. (2009). A human stem cell-based model for identifying adverse effects of organic and inorganic chemicals on the developing nervous system. Stem Cells.

[20] Grandjean P., Weihe P., Debes F., Choi A.L., Budtz-Jørgensen E. (2014). Neurotoxicity from prenatal and postnatal exposure to methylmercury. Neurotoxicol. Teratol..

[21] Koh A.S., Simmons-Willis T.A., Pritchard J.B., Grassl S.M., Ballatori N. (2002). Identification of a mechanism by which the methylmercury antidotes N-acetylcysteine and dimercaptopropanesulfonate enhance urinary metal excretion: Transport by the renal organic anion transporter-1. Mol. Pharmacol..

[22] Falluel-Morel A., Lin L., Sokolowski K., McCandlish E., Buckley B., DiCicco-Bloom E. (2012). N-acetyl cysteine (NAC) treatment reduces mercury-induced neurotoxicity in the developing rat hippocampus. J. Neurosci. Res..

[23] Yuntao F., Chenjia G., Panpan Z., Wenjun Z., Suhua W., Guangwei X., Haifeng S., Jian L., Wanxin P., Yun F. (2016). Role of autophagy in methylmercury-induced neurotoxicity in rat primary astrocytes. Arch. Toxicol..

[24] Myers G.J., Thurston S.W., Pearson A.T., Davidson P.W., Cox C., Shamlaye C.F., Cernichiari E., Clarkson T.W. (2009). Postnatal exposure to methyl mercury from fish consumption: A review and new data from the Seychelles Child Development Study. Neurotoxicology.

[25] Ehrenstein C., Shu P., Wickenheiser E., Hirner A., Dolfen M., Emons H., Obe G. (2002). Methyl mercury uptake and associations with the induction of chromosomal aberrations in Chinese hamster ovary (CHO) cells. Chem. -Biol. Interact..

[26] Lin T., Ruan S., Huang D., Meng X., Li W., Wang B., Zou F. (2019). MeHg-induced autophagy via JNK/Vps34 complex pathway promotes autophagosome accumulation and neuronal cell death. Cell Death Dis..

[27] Liu X., Wang J., Yang Z., Xie Q., Diao X., Yao X., Huang S., Chen R., Zhao Y., Li T. (2024). Upregulated DNMT3a coupling with inhibiting p62-dependent autophagy contributes to NNK tumorigenicity in human bronchial epithelial cells. Ecotoxicol. Environ. Saf..

[28] Baumgardner J.N., Shankar K., Hennings L., Albano E., Badger T.M., Ronis M.J.J. (2008). N-Acetylcysteine Attenuates Progression of Liver Pathology in a Rat Model of Nonalcoholic Steatohepatitis. J. Nutr..

[29] Su Z.-Y., Khor T.O., Shu L., Lee J.H., Saw C.L.-L., Wu T.-Y., Huang Y., Suh N., Yang C.S., Conney A.H. (2013). Epigenetic reactivation of Nrf2 in murine prostate cancer TRAMP C1 cells by natural phytochemicals Z-ligustilide and Radix angelica sinensis via promoter CpG demethylation. Chem. Res. Toxicol..

[30] Hou S., Wang C., Ma X., Zhao J., Wang J., Fang Y., Liu H., Ding H., Guo J., Lu W. (2025). Methylmercury Chloride Exposure Affects Oocyte Maturation Through AMPK/mTOR-Mediated Mitochondrial Autophagy. Int. J. Mol. Sci..

[31] Azad G.K., Singh V., Mandal P., Singh P., Golla U., Baranwal S., Chauhan S., Tomar R.S. (2014). Ebselen induces reactive oxygen species (ROS)-mediated cytotoxicity in Saccharomyces cerevisiae with inhibition of glutamate dehydrogenase being a target. FEBS Open Bio.

[32] Ballatori N., Lieberman M.W., Wang W. (1998). N-acetylcysteine as an antidote in methylmercury poisoning. Environ. Health Perspect..

[33] Shanker G., Syversen T., Aschner J.L., Aschner M. (2005). Modulatory effect of glutathione status and antioxidants on methylmercury-induced free radical formation in primary cultures of cerebral astrocytes. Mol. Brain Res..

[34] Sumi D. (2008). Biological Effects of and Responses to Exposure to Electrophilic Environmental Chemicals. J. Health Sci..

[35] Feng S., Xu Z., Wang F., Yang T., Liu W., Deng Y., Xu B. (2017). Sulforaphane Prevents Methylmercury-Induced Oxidative Damage and Excitotoxicity Through Activation of the Nrf2-ARE Pathway. Mol. Neurobiol..

[36] da Rosa-Silva H.T., Panzenhagen A.C., Espitia-Pérez P., Teixeira A.A., Roitman A., Almeida R.F., Heimfarth L., Moreira J.C.F. (2020). Effects of foetal and breastfeeding exposure to methylmercury (MeHg) and retinol palmitate (Vitamin A) in rats: Redox parameters and susceptibility to DNA damage in liver. Mutat. Res..

[37] Aremu D.A., Madejczyk M.S., Ballatori N. (2008). N-acetylcysteine as a potential antidote and biomonitoring agent of methylmercury exposure. Environ. Health Perspect..

[38] Palmer L.A., Doctor A., Chhabra P., Sheram M.L., Laubach V.E., Karlinsey M.Z., Forbes M.S., Macdonald T., Gaston B. (2007). S-nitrosothiols signal hypoxia-mimetic vascular pathology. J. Clin. Investig..

[39] Hildebrandt W., Alexander S., Bärtsch P., Dröge W. (2002). Effect of N-acetyl-cysteine on the hypoxic ventilatory response and erythropoietin production. Blood.

[40] Chang J., Yang B., Zhou Y., Yin C., Liu T., Qian H., Xing G., Wang S., Li F., Zhang Y. (2019). Acute methylmercury exposure and the hypoxia-inducible factor-1a signaling pathway under normoxic conditions in the rat brain and astrocytes in vitro. Environ. Health Perspect..

[41] Muniroh M., Gumay A.R., Indraswari D.A., Bahtiar Y., Hardian H., Bakri S., Maharani N., Karlowee V., Koriyama C., Yamamoto M. (2020). Activation of MIP-2 and MCP-5 Expression in Methylmercury-Exposed Mice and Their Suppression by N-Acetyl-L-Cysteine. Neurotox. Res..

[42] Lin X., Wei M., Song F., Xue D., Wang Y. (2020). N-acetylcysteine (NAC) attenuating apoptosis and autophagy in RAW264.7 cells. Pol. J. Microbiol..

[43] Fan J., Ren D., Wang J., Liu X., Zhang H., Wu M., Yang G. (2020). Bruceine D induces lung cancer cell apoptosis and autophagy via the ROS/MAPK signaling pathway. Cell Death Dis..

[44] El-Sherbeeny N.A., Soliman N., Youssef A.M., Abd El-Fadeal N.M., El-Abaseri T.B., Hashish A.A. (2020). The protective effect of biochanin A against rotenone-induced neurotoxicity in mice involves enhancing of PI3K/Akt/mTOR signaling and beclin-1 production. Ecotoxicol. Environ. Saf..

[45] Pi H., Li M., Zou L., Yang M., Deng P., Fan T., Liu M., Tian L., Tu M., Xie J. (2019). AKT inhibition-mediated dephosphorylation of TFE3 promotes overactive autophagy independent of MTORC1 in cadmium-exposed bone mesenchymal stem cells. Autophagy.

[46] Chang S.-H., Lee H.J., Kang B., Yu K.-N., Minai-Tehrani A., Lee S., Kim S.U., Cho M.-H. (2013). Methylmercury induces caspase-dependent apoptosis and autophagy in human neural stem cells. J. Toxicol. Sci..

[47] Shimizu E., Hashimoto K., Komatsu N., Iyo M. (2002). Roles of endogenous glutathione levels on 6-hydroxydopamine-induced apoptotic neuronal cell death. Neuropharmacology.

[48] Clarkson T.W., Vyas J.B., Ballatori N. (2007). Mechanisms of mercury disposition in the body. Am. J. Ind. Med..

[49] Madejczyk M.S., Aremu D.A., Simmons-Willis T.A., Clarkson T.W., Ballatori N. (2007). Accelerated urinary excretion of methylmercury following administration of its antidote N-acetylcysteine requires Mrp2/Abcc2, the apical multidrug resistance-associated protein. J. Pharmacol. Exp. Ther..

[50] Heggland I., Kaur P., Syversen T. (2023). Uptake and efflux of methylmercury in vitro: Comparison of transport mechanisms in C6, B35 and RBE4 cells. Toxicol. Vitr..

[51] Rooney J.P.K. (2007). The role of thiols, dithiols, nutritional factors and interacting ligands in the toxicology of mercury. Toxicology.

[52] Zalups R.K., Ahmad S. (2005). Transport of N-acetylcysteine s-conjugates of methylmercury in Madin-Darby canine kidney cells. J. Pharmacol. Exp. Ther..

